# Supplementary material for: Arginine π-stacking drives binding to fibrils of the Alzheimer protein Tau
Source: Nat Commun. 2020 Jan 29;11:571. doi: 10.1038/s41467-019-13745-7 (PMC6989696; doi:10.1038/s41467-019-13745-7)
Supplement: Supplementary file 1 — Supplementary Information [file 41467_2019_13745_MOESM1_ESM.pdf]

Arginine π-stacking drives binding to fibrils of the Alzheimer protein Tau

SUPPLEMENTARY INFORMATION

Luca Ferrari, Riccardo Stucchi *et al.*

SUPPLEMENTARY TABLES

Supplementary Table 1. List of primers used in this study

Primer sequence 5'>3'	***Note***
**AGGTCTCGGGTGGT**GATTACAAGGATGACGACGATAAG**CAGACCGCTCCGGTGCCG**	Forward primer used to subclone Tau-RD* into a pET24 plasmid modified with a His$_6$-Smt-tag upstream of insertion site.
**TGCTCTCGAGTTATTCGATTTTTTTGTTTCC**	Reverse primer used to subclone Tau-RD* into a pET24 plasmid modified with a His$_6$-Smt-tag upstream of insertion site.
**AGGTCTCGGGTGGTATGTATCCGTATGATGTGCCGGATTATGCGATGGCGGAGCAGGGAG**	Forward primer used to subclone Map7 truncations into a pET24 plasmid modified with a His$_6$-Smt-tag upstream of insertion site.
**TGCTAAGCTTTCAAACAACGCTGCTCTCCCAGG**	Reverse primer used to subclone Map7 truncations into a pET24 plasmid modified with a His$_6$-Smt-tag upstream of insertion site.

Supplementary Table 2. Additional reagents and software information

REAGENT or RESOURCE	***SOURCE***	***IDENTIFIER***
Antibodies		
Anti-FLAG M2 mouse monoclonal antibody	Sigma-Aldrich	F1804
Anti-HA 12CA5 mouse hybridoma	Sigma-Aldrich	ROAHA – Anti-HA (12CA5)
Anti-FLAG® M2 Magnetic Beads	Sigma-Aldrich	M8823
Donkey anti Mouse IgG Alexa Fluor 680	Invitrogen	A10038
Donkey anti Mouse IgG IR Dye 800 conjugated	Rockland	610-732-002
Goat anti-rabbit IR Dye 800nm conjugated	Rockland	611-132-002
Bacterial and Virus Strains		
BL21 Rosetta 2 cells	Novagen	71402
peT24a	Novagen	69749

peT23a	Novagen	69745
Chemicals, Peptides, and Recombinant Proteins		
4x Laemmli Sample Buffer	Biorad	161-0747
Acetic acid 100%	Merck	64-19-7
Acetonitrile	Merck	100030
Albumin from chicken egg	Sigma-Aldrich	A7641
Ammonium bicarbonate	Sigma-Aldrich	09830
Ampicillin Sodium Salt	Sigma-Aldrich	A9518
Chloramphenicol	Sigma-Aldrich	C0378
cOmplete Protease cocktail inhibitors	Roche	CO-RO
cOmplete Protease EDTA free cocktail inhibitors	Roche	COEDTAF-RO
DTT	Thermo Fischer Scientific	R0862
GelCode Blue Stain Reagent	Thermo Fischer Scientific	24590
Heparin low Molecular Weigth	Santa Cruz Biotech	1-8-9041
HEPES	Sigma-Aldrich	H3375
HEPES sodium salt	Sigma-Aldrich	H7006
Imidazole	Sigma-Aldrich	56749
IPTG	Thermo Fischer Scientific	15529019
Kanamycin Sulfate from Streptomyces kanamyceticus	Sigma-Aldrich	K4000
KCl	Carl Roth	6781.2
LI-COR Odyssey Blocking Buffer (TBS)	Westburg	LI 927-50000
Imidazole	Sigma-Aldrich	56749
Iodoacetamide	Merck	144-48-9
Methanol	Merck	106009
NaCl	Carl Roth	HN00.3
Nonidet® P40	AppliChem	A1694
SDS Solution, 20 %	Serva	20767.03
Sodium Azide	Sigma-Aldrich	S8032
Sodium Fluoride	Sigma-Aldrich	S7920
Sodium phosphate monobasic	Sigma-Aldrich	S3139
Sucrose	Sigma-Aldrich	S0389
Thioflavin T	Sigma-Aldrich	2390-54-7
Triton X-100	Sigma-Aldrich	9002-93-1
Trizma base	Sigma-Aldrich	T6066
Trypsin	Promega	V5111
Ulp1	In-house	NA
β-mercaptoethanol	Sigma-Aldrich	M3148
Critical Commercial Assays		
BCA assay	Thermo Fischer Scientific	23250

Wizard Plus SV Minipreps	Promega	A1460
Recombinant DNA		
Pet24a human SUMO-Tau-RD*	In-house	NA
peT24a mouse MAP7 Smt6-HA-M1-S227 wt	In-house	NA
peT24a mouse MAP7 Smt6-HA-M1-S227 wt	In-house	NA
Pet23a human HisHsp90β	In-house	NA
Software and Algorithms		
Crapome	www.crapome.org	NA
ImageJ	https://imagej.nih.gov/ij/	
Image Studio 2.10.1	LI-COR	NA
Perseus	https://www.nature.com/articles/nmeth.3901#plugin-architecture	NA
Prism7	Graphpad	NA
Proteome Discoverer 1.4	ThermoScientific	NA
R	https://www.r-project.org/	NA
Xcalibur™ Software	ThermoScientific	NA
Other		
4–12% Criterion XT Bis-Tris Protein Gel	Biorad	3450123
96-Well Polypropylene Deep Well Storage Plates	Thermo Fischer Scientific	260251
96-Well Greiner plates, Polystyrene	Sigma-Aldrich	M3061
Agilent 1290 Infinity LC	Agilent Technologies	
Circular Dichroism spectropolarimeter J-810	JASCO	0302-0407A
DynaMag -2 Magnet	Invitrogen	10723874
HiTrap heparin column High Performance	GE Healthcare	17-0407-01
POROS20HQ anion exchange column	Thermo Fischer Scientific	1232906
POROS20HQ cation exchange column	Thermo Fischer Scientific	1332906
POROS20MC affinity purification column	Thermo Fischer Scientific	1542906
Poroshell 120 EC C18 columns	Agilent Technologies	699775-922
ND-1000 UV/Vis spectrophotometer	NanoDrop Technologies	S137007
Q Exactive Orbitrap Mass Spectrometer	Thermo Fischer Scientific	IQLAAEGAA PFALGMAZR
Reprosil-Pur Basic C18, 3 μm	Dr.Maisch	r13.b9.
Spectra/Por Dialysis Membrane MWCO 12-14 kDa	SpectrumLaboratories	132703
Spectra/Por Dialysis Membrane MWCO 3.5 kDa	SpectrumLaboratories	132724

SpectraMax i3	Molecular Devices	NA
Sorvall WX Ultra Series Centrifuge WX80	Thermo Fischer Scientific	75000080
Vivaspin 6 buffer exhange filter	Sartorius	VS0612
Whatman Protran Nitrocellulose membrane	Sigma-Aldrich	Z670952

SUPPLEMENTARY FIGURES

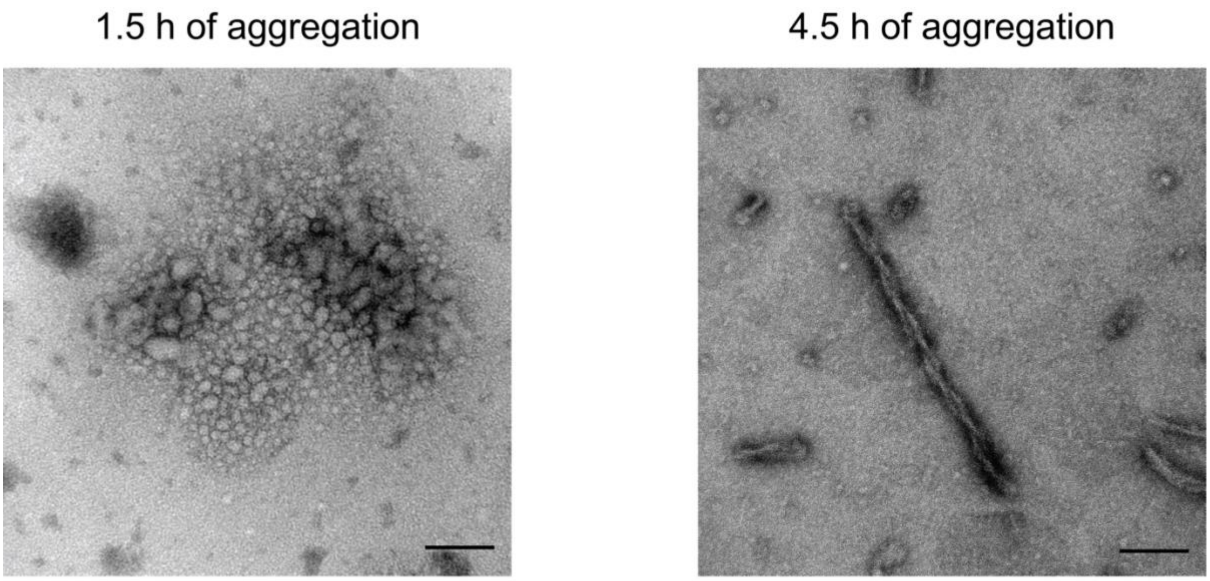

Supplementary Figure 1. Additional TEM images of aggregating Tau-RD*

Transmission Electron Microscopy of Tau-RD* fibrils, timepoint 1.5 and 4.5 h. Scalebar 100 nm.

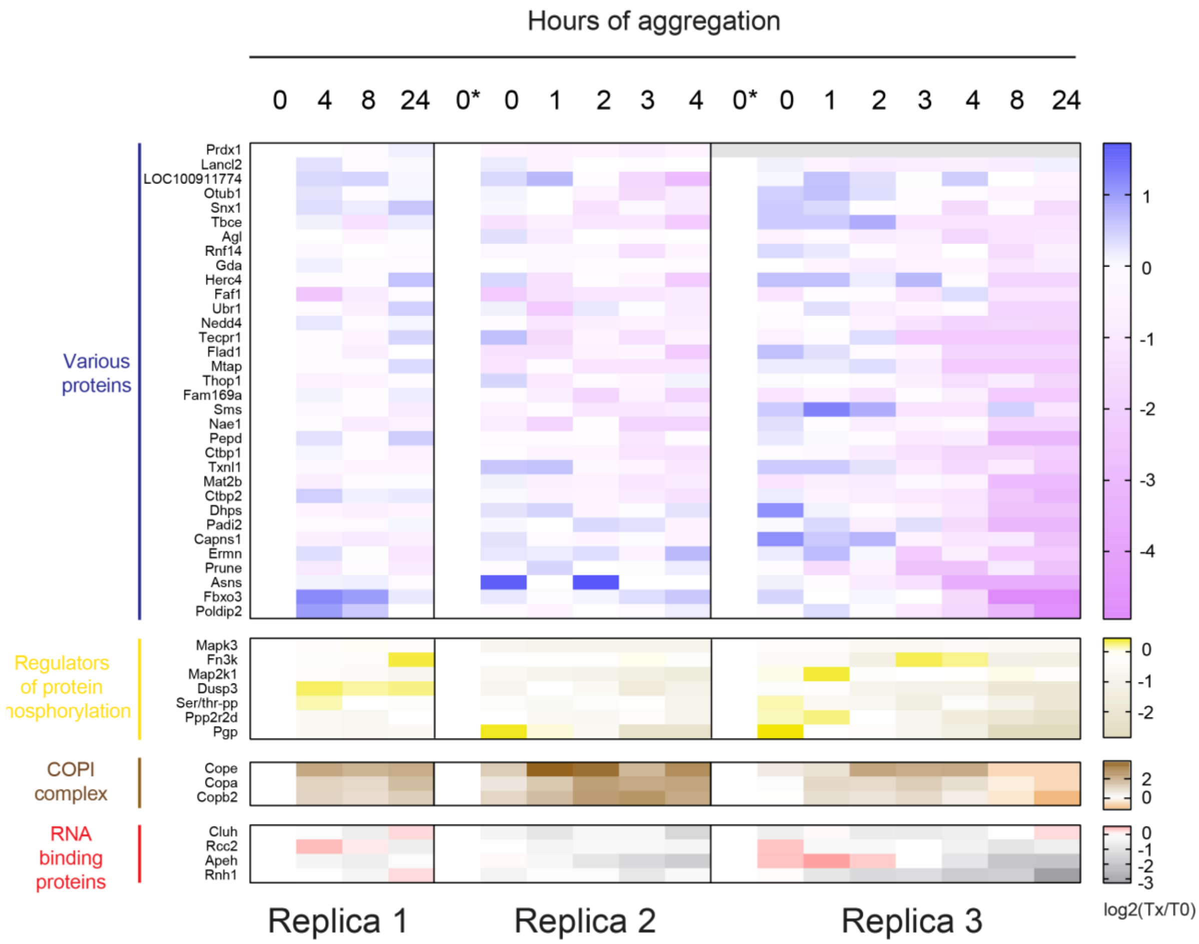

Supplementary Figure 2. Interactome rewiring of monomeric-specific interactors, 3 independent biological replicates.

Heat map showing an unbiased selection of Tau-RD* lost proteins upon aggregation. Only proteins with a Fold Change calculation > 2 (FC-A, Crapome; by averaging the spectral counts across the selected controls) were considered enriched in Tau-RD* monomers compared to control empty beads in the 3 different replicates.

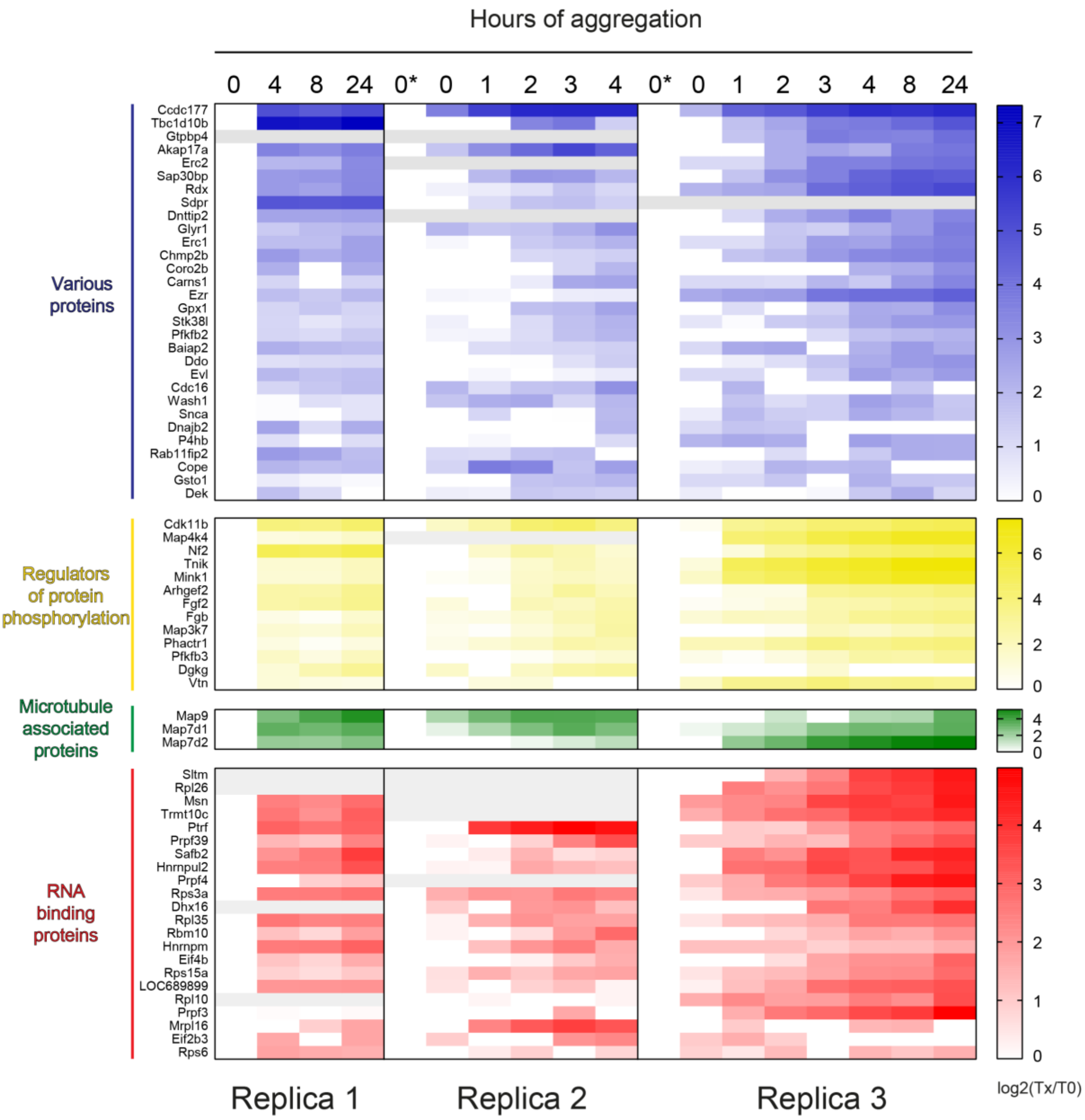

Supplementary Figure 3. Interactome rewiring of fibrillar-specific interactors, 3 independent biological replicates.

Heat map showing an unbiased selection of Tau-RD* sequestered proteins upon aggregation. Only proteins with a Fold Change calculation > 2 (FC-A, Crapome; by averaging the spectral counts across the selected controls) were considered enriched in Tau-RD* aggregates compared to Tau-RD* monomers in the 3 different replicates.

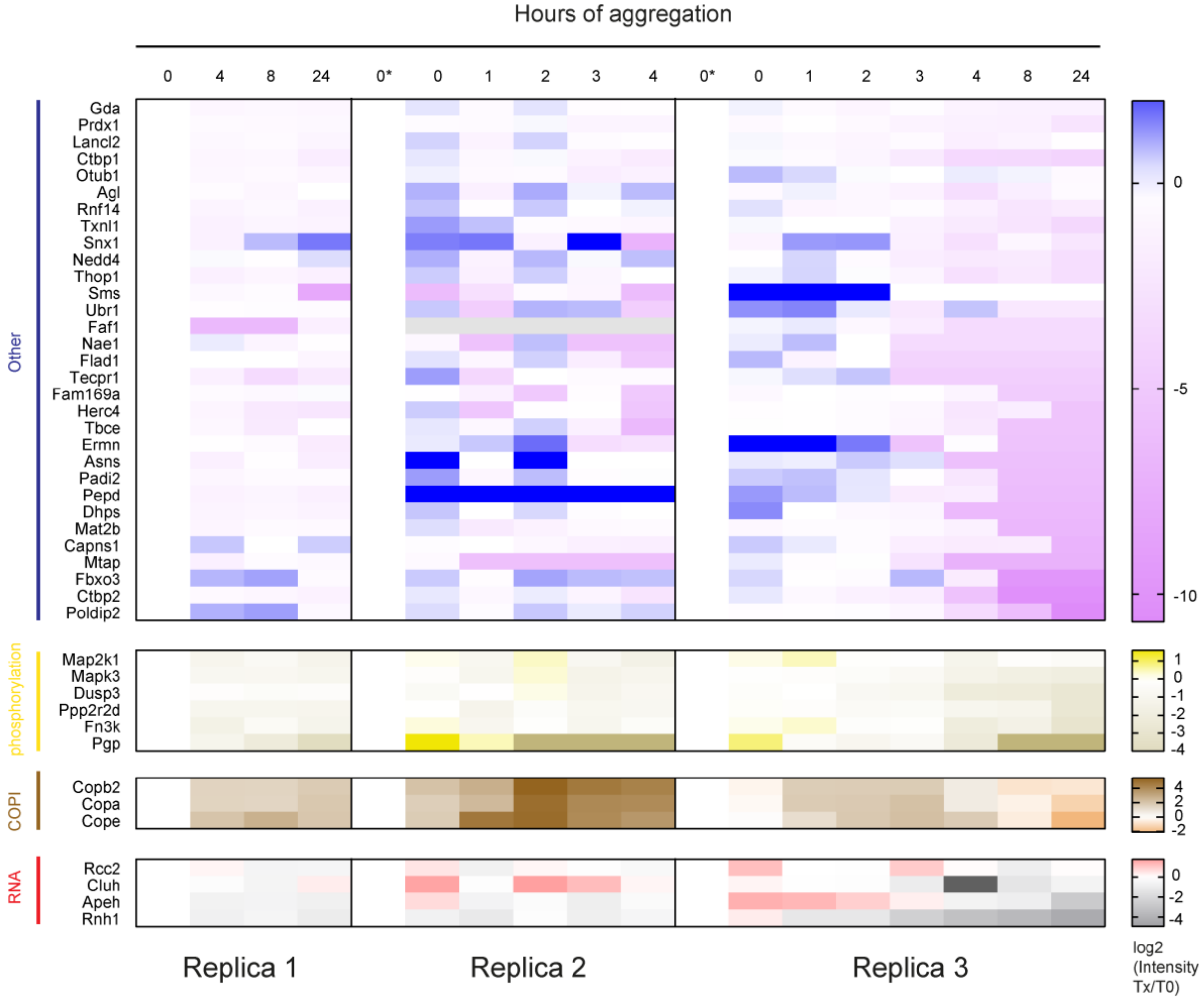

Supplementary Figure 4. Interactome rewiring of monomeric-specific interactors, 3 independent biological replicates.

Intensity based quantification was used instead of PSMs counting.

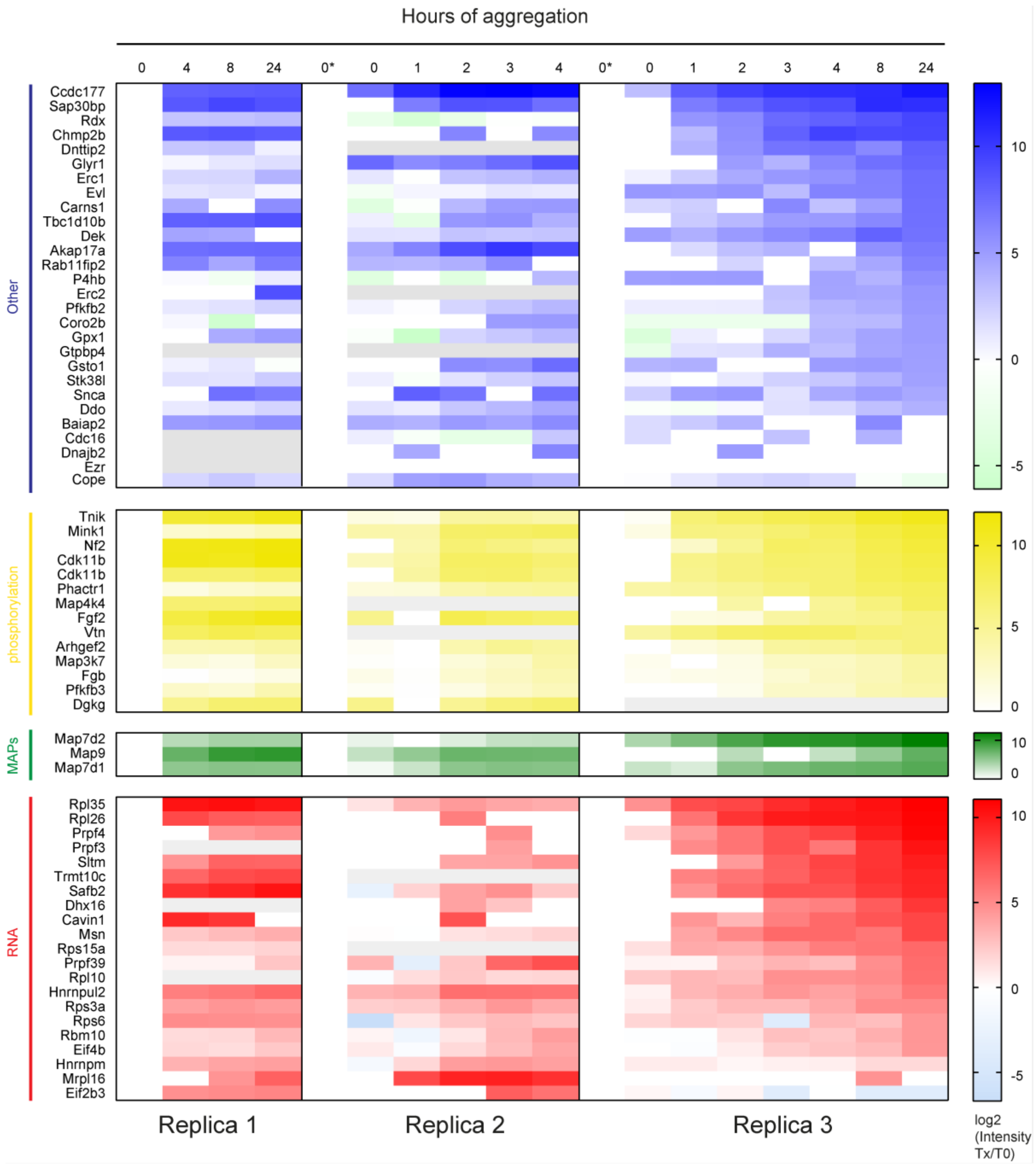

Supplementary Figure 5. Interactome rewiring of fibrillar-specific interactors, 3 independent biological replicates.

Intensity based quantification was used instead of PSMs counting.

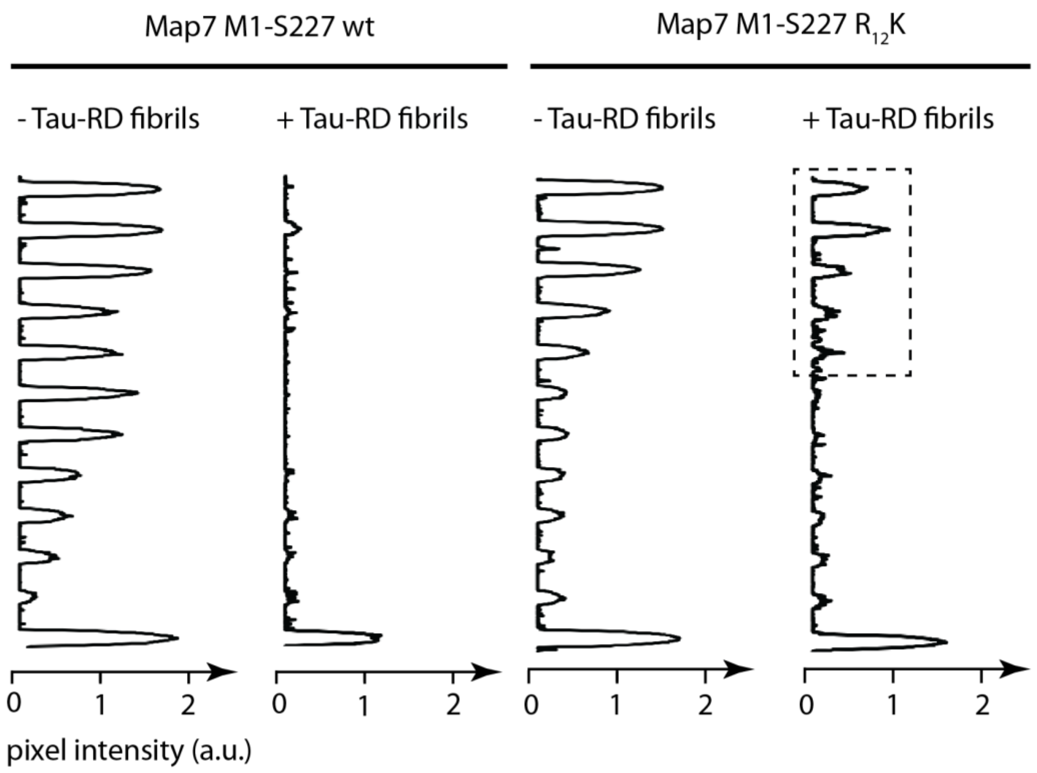

Supplementary Figure 6. Quantification of anti-HA blot.

Profile of anti-HA dot blots, both for Map7 M1-S-227 wt and $R_{12}K$, Dashed box indicates Map7 M1-S-227 $R_{12}K$ not associated to Tau-RD* fibrils.

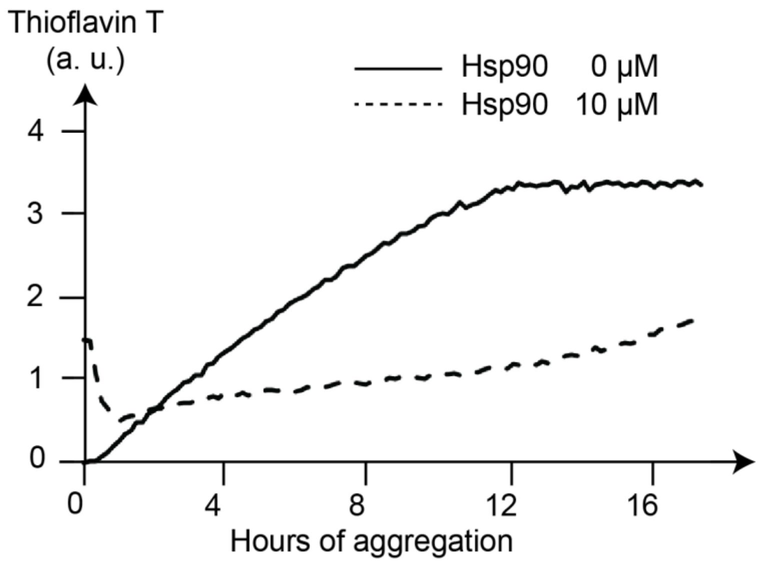

Supplementary Figure 7. Hsp90 blocks Tau aggregation

Repetition of **Fig. 5A**. Thioflavin T assay to detect decrease of amyloid content in the presence of Hsp90 10 μM. Fluorescence is expressed as arbitrary unit (a. u.).

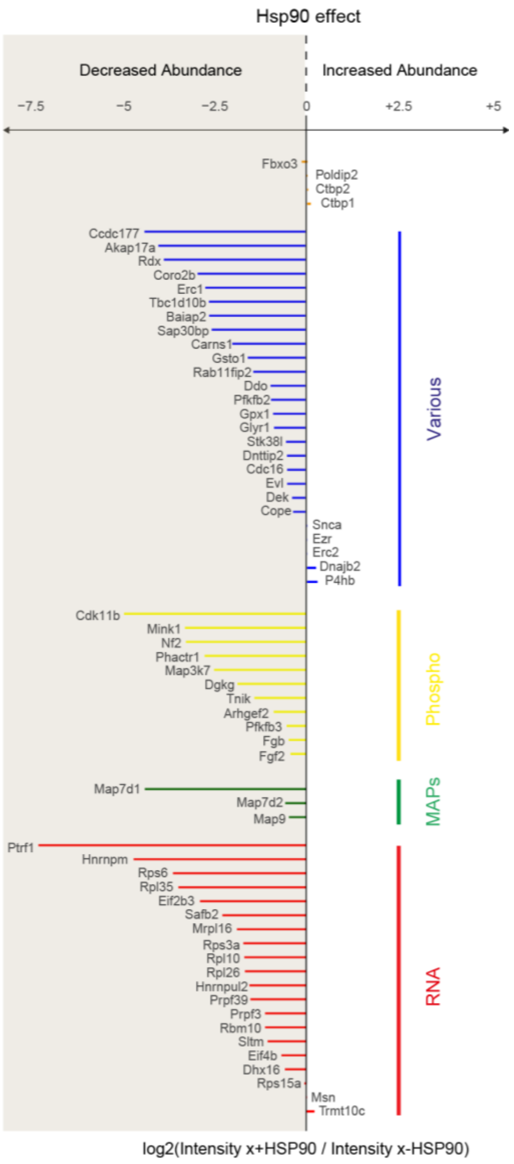

Supplementary Figure 8. Hsp90 remodels Tau aggregation-specific interactome.

Intensity based quantification was used instead of PSMs counting.

Figure 1A

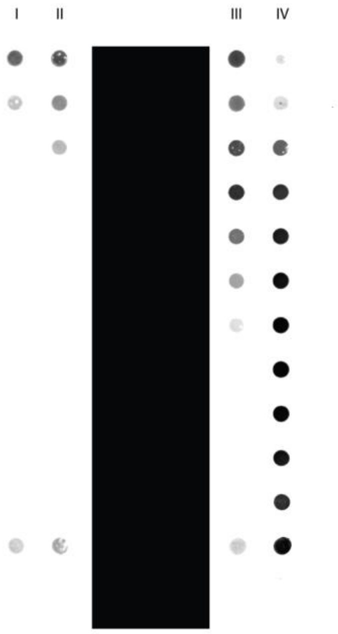

Figure 4D

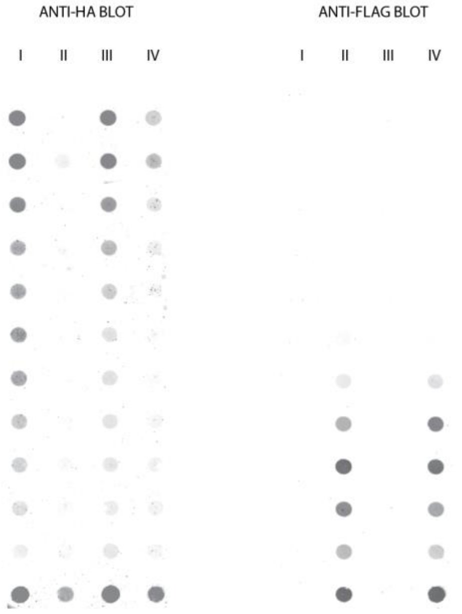

Figure 5B

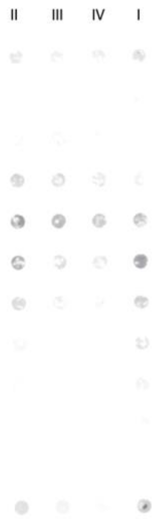

Supplementary Figure 9. Uncropped gels

All uncropped blots presented in this study. Each tube is labelled as in the main text.
