# Supplementary material for: Arginine π-stacking drives binding to fibrils of the Alzheimer protein Tau
Source: Nat Commun. 2020 Jan 29;11:571. doi: 10.1038/s41467-019-13745-7 (PMC6989696; doi:10.1038/s41467-019-13745-7)
Supplement: Supplementary file 3 — Description of Additional Supplementary Files [file 41467_2019_13745_MOESM3_ESM.pdf]

Description of Additional Supplementary Files

File name: Supplementary Data 1

Description: PSMs based quantifications of MS-identified proteins in Tau-RD* aggregates. Related to Figure 2 and Supplementary Figures 2-3.

File name: Supplementary Data 2

Description: Intensity based quantifications of MS-identified proteins in Tau-RD* aggregates. Related to Supplementary Figures 4-5.

File name: Supplementary Data 3

Description: PSMs based quantifications of MS-identified proteins in Tau-RD* fibrils in the presence/absence of Hsp90. Related to Figure 6.

File name: Supplementary Data 4

Description: Intensity based quantifications of MS-identified proteins in Tau-RD* fibrils in the presence/absence of Hsp90. Related to Supplementary Figure 8.
